# Predict Early Recurrence of Resectable Hepatocellular Carcinoma Using Multi-Dimensional Artificial Intelligence Analysis of Liver Fibrosis

**DOI:** 10.3390/cancers13215323

**Published:** 2021-10-23

**Authors:** I-Ting Liu, Chia-Sheng Yen, Wen-Lung Wang, Hung-Wen Tsai, Chang-Yao Chu, Ming-Yu Chang, Ya-Fu Hou, Chia-Jui Yen

**Affiliations:** 1 Institute of Clinical Medicine, College of Medicine, National Cheng Kung University, Tainan 70401, Taiwan; tim1226.tw@yahoo.com.tw; 2 Department of Oncology, National Cheng Kung University Hospital, College of Medicine, National Cheng Kung University, Tainan 70403, Taiwan; wwenlung@gmail.com (W.-L.W.); myc1209@gmail.com (M.-Y.C.); yafu0815@gmail.com (Y.-F.H.); 3 Division of General Surgery, Department of Surgery, Kaohsiung Veterans General Hospital, Kaohsiung 81362, Taiwan; gsvsycs@gmail.com; 4 Department of Pathology, National Cheng Kung University Hospital, College of Medicine, National Cheng Kung University, Tainan 70403, Taiwan; hungwen@mail.ncku.edu.tw; 5 Department of Pathology, Chi-Mei Medical Center, Tainan 71004, Taiwan; b00804@mail.chimei.org.tw

**Keywords:** liver fibrosis, hepatocellular carcinoma, recurrence, SHG/TPEF microscopy, artificial intelligence

## Abstract

**Simple Summary:**

Hepatocellular carcinoma (HCC) is the third most commonly diagnosed cancer in the world, and surgical resection is the commonly used curative management of early-stage disease. However, the recurrence rate is high after resection, and liver fibrosis has been thought to increase the risk of recurrence. Conventional histological staging of fibrosis is highly subjective to observer variations. To overcome this limitation, we used a fully quantitative fibrosis assessment tool, qFibrosis (utilizing second harmonic generation and two-photon excitation fluorescence microscopy), with multi-dimensional artificial intelligence analysis to establish a fully-quantitative, accurate fibrotic score called a “combined index”, which can predict early recurrence of HCC after curative intent resection. Therefore, we can pay more attention on the patients with high risk of early recurrence.

**Abstract:**

Background: Liver fibrosis is thought to be associated with early recurrence of hepatocellular carcinoma (HCC) after resection. To recognize HCC patients with higher risk of early recurrence, we used a second harmonic generation and two-photon excitation fluorescence (SHG/TPEF) microscopy to create a fully quantitative fibrosis score which is able to predict early recurrence. Methods: The study included 81 HCC patients receiving curative intent hepatectomy. Detailed fibrotic features of resected hepatic tissues were obtained by SHG/TPEF microscopy, and we used multi-dimensional artificial intelligence analysis to create a recurrence prediction model “combined index” according to the morphological collagen features of each patient’s non-tumor hepatic tissues. Results: Our results showed that the “combined index” can better predict early recurrence (area under the curve = 0.917, sensitivity = 81.8%, specificity = 90.5%), compared to alpha fetoprotein level (area under the curve = 0.595, sensitivity = 68.2%, specificity = 47.6%). Using a Cox proportional hazards analysis, a higher “combined index” is also a poor prognostic factor of disease-free survival and overall survival. Conclusions: By integrating multi-dimensional artificial intelligence and SHG/TPEF microscopy, we may locate patients with a higher risk of recurrence, follow these patients more carefully, and conduct further management if needed.

## 1. Introduction

Hepatocellular carcinoma (HCC) is the fourth most common cause of cancer deaths around the world [1]. It is also the fifth most commonly diagnosed cancer and is the second most common cause of cancer deaths in Taiwan [2]. The majority of HCC (75–80%) cases are attributable to persistent viral infections with the hepatitis B virus (HBV) (50–65%) and hepatitis C virus (HCV) (10–15%) in Taiwan [3]. Carcinogenesis of HCC is a very complex multi-factor process, including viral or non-viral causes such as alcoholic hepatitis and nonalcoholic steatohepatitis (NASH) [4]. Chronic hepatitis infection causes liver inflammation and damage, subsequent fibrosis, and liver regeneration that may lead to malignant transformation of the liver [5]. In early-stage HCC, potentially curative treatments are available. They include surgical resection, percutaneous ablation, and liver transplantation. Percutaneous ablation and liver transplantation can only be applied in carefully selected patients depending on the patient’s tumor status and general condition as well donor availability. Therefore, surgical resection is the most commonly used curative management of HCC. However, the recurrence rate is high after resection, especially within the first two years [6]. About 50% to 90% of postoperative deaths after curative resection are a result of recurrence of the disease, and intrahepatic recurrence accounts for the majority of cases. Liver fibrosis has been thought to increase the risk of intrahepatic recurrence after hepatectomy in the case of HCC [7].

Conventional histological staging of fibrosis, such as the Ishak fibrotic score, is highly subjective and prone to sampling error and observer variations. Second harmonic generation and two-photon microscopy was first used as a comprehensive, morphology-based, quantified method for scoring liver fibrosis [8,9,10]. qFibrosis uses a system of second harmonic generation plus two-photon excitation fluorescence (SHG/TPEF) microscopy to image tissue samples and establish an index by (i) identification of different collagen patterns, (ii) extraction of collagen architectural features, and (iii) statistical analysis of features of the respective collagen patterns. qFibrosis scoring has been analyzed employing Metavir and Ishak fibrosis staging as standard references and has been established as a fully-quantitative, innovative method incorporating histological features to facilitate accurate fibrosis scoring in animal models and chronic hepatitis B patients [11]. Besides this, it was also applied to quantitatively identify subtle changes of liver fibrosis in chronic hepatitis B patients following antiviral therapy as well as to accurately assess fibrosis in non-alcoholic fatty liver disease patients in more recent studies [12,13,14]. Therefore, this study involves the use of this more accurate fibrosis scoring method to evaluate the fibrotic status of the hepatic tissue of patients with HCC after hepatectomy.

The application of qFibrosis is intended enable the prediction of early recurrence after curative intent hepatectomy according to the fibrotic features of hepatic tissue. Thus, patients identified as high-risk for early recurrence can be followed more carefully in shorter intervals following hepatectomy. In this study, we generated a “combined index” using multi-dimensional artificial intelligence analysis of qFibrosis with the features of fibrosis from 81 patients receiving partial hepatectomy. When the combined index is larger than 0.501, early recurrence is more likely.

## 2. Materials and Methods

### 2.1. Study Population

Adult patients who were diagnosed and staged by liver tumor biopsy, abdomen triphasic computed tomography (CT), and alpha fetoprotein (AFP) as resectable HCC with known HBV or HCV infection and planning to have curative intent surgical resection were enrolled in this study. Patients with co-infection of HBV and HCV, inadequate tissue samples or history of other malignancy within 2 years prior to screening were excluded (detailed inclusion and exclusion criteria as Table S1). These patients receive regular follow-up with abdomen triphasic CT after surgery. Informed consent regarding use of tissue samples, clinical data, and medical records for this research was obtained from all enrolled patients. The clinical and pathological staging used in this study was The American Joint Committee on Cancer (AJCC) 7th edition. All experimental protocols and study methods conformed to the ethical guidelines of the Declaration of Helsinki and were approved by the Institutional Review Board of Human Research at National Cheng Kung University Hospital and Chi Mei Medical Center. 

### 2.2. Image Acquisition System

Images were acquired on unstained sections of non-tumor liver samples, using a Genesis (HistoIndex Pte. Ltd, Singapore) system, in which second harmonic generation (SHG) microscopy was used to visualize collagen, and the other cell structures were visualized using two-photon excited fluorescence (TPEF) microscopy. 

The samples were laser-excited at 780 nm; SHG signals were recorded at 390 nm, and TPEF signals were recorded at 550 nm. Image acquisition was performed at a 20× magnification for each 200 × 200 μm2 image. Multiple adjacent images were captured to encompass large areas. To cover most of the sample areas, 10 five-by-five multi-tile images were acquired for each human sample, with a final image size of 10 mm^2^ (10 × 1 × 1 mm).

### 2.3. Image Quantification

Total collagen percentages and other collagen features, including specific collagen strings and collagen connectivity-related measurements, were used to predict early recurrence (disease free (DF) < 1 year) and late recurrence (DF ≥ 1 years) post operation HCC.

**Figure 1:**
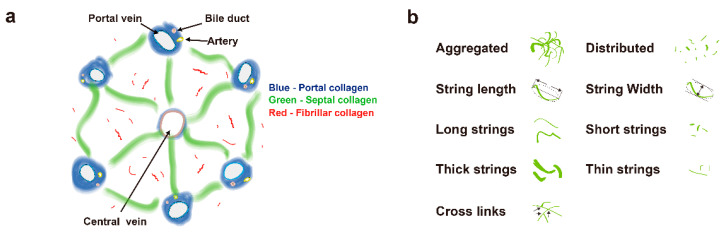
Schematic illustration of the studied collagen features for the prediction of early recurrence. (**a**) Representation of collagen in portal, septal, and fibrillar regions, which are denoted in blue, green, and red, respectively. (**b**) Representation of some features of collagen strings.

**Figure 2:**
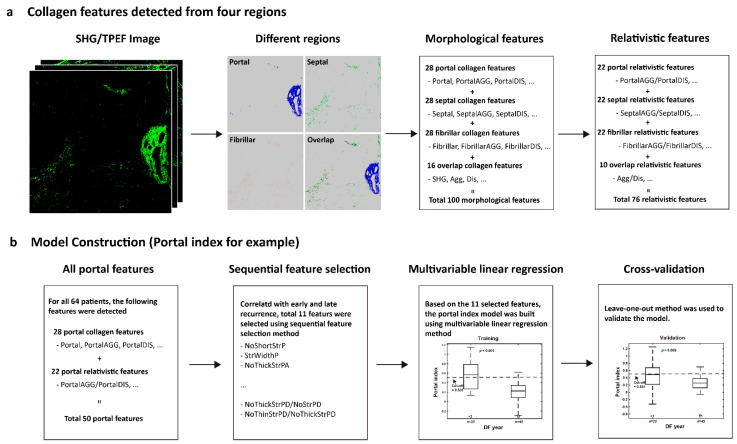
Flowchart of model construction. (**a**) Total 100 morphological features were detected from portal, septal, fibrillar, and overlap regions. Another 76 relativistic features were constructed based on the morphological features. (**b**) The method of portal index is for example. Sequential feature selection method was performed to reduce the dimensionality of data by selecting only a subset of collagen features. A total of 11 features were selected to build the model using multivariable linear regression method. To validate the prediction model, leave-one-out cross-validation method was used. The methods for septa index, fibrillar index, overlap index, and combined index are similar. For combined index, a total of 18 features were selected from 176 features to build the model.

A total of 100 morphological features were initially used in this study. Collagen in the overall region was classified into three specific areas: portal collagen (portal expansion), septal collagen (bridging fibrosis), and fibrillar collagen (fine collagen distributed in the pericellular/perisinusoidal space) [11]. Furthermore, in addition to the total measures, collagen was also measured in two different patterns, namely, distributed collagen (fine collagen) and aggregated collagen (large patches). For each pattern in these specific regions (portal, septal, and fibrillar), collagen strings were categorized into short strings, long strings, thin strings, and thick strings according to string length and width (Figure 1a,b). Based on the 100 collagen morphological features, another 76 relativistic features were constructed. Each relativistic feature was the ratio of two morphological features, such as the ratio of the number of short strings to the number of long strings (NoShortStr/NoLongStr) and the ratio of aggregated collagen to distributed collagen (AGG/DIS). Thus, total 176 features were used for model construction (Figure 2a).

### 2.4. Model Construction

To predict early recurrence in patients with hepatocellular carcinoma after curative hepatectomy, a prediction model was developed based on the quantified collagen features. 

Firstly, each feature was normalized to a value between 0–1 according to its maximum and minimum values. Secondly, feature selection was performed to reduce the dimensionality of data by selecting only a subset of collagen features. A common method of feature selection, named sequential feature selection was used in this study [15]. In the procedure of sequential feature selection, a linear regression model was used whereby the criterion was the residual sum of squares and the search algorithm was sequential forward selection. In total, 64 cases with HBV or HCV but no NASH were used to find the most significant collagen features related with early recurrence. 

Next, a model was trained to predict early recurrence in patients with hepatocellular carcinoma after curative hepatectomy using a “combined index”, which was constructed from the previously mentioned 64 cases with multivariable linear regression method. To validate the prediction model, leave-one-out cross-validation method was used [16,17]. Briefly, one sample is randomly retained as the validation data while the remaining 63 cases are used as training data to construct the model. The performance of the prediction model is then tested on the single validation case. The cross-validation process is repeated 64 times, with a different case left out each time. The data of combined index for statistical analysis in the study, in the absence of special note was the prediction values by leave-one-out cross-validation method.

Thus, for each HCC patient after hepatectomy, a combined index can be calculated on the SHG/TPEF image using the recurrence prediction model (Figure 2b). This feature indicates that a higher value of the combined index correlates with early recurrence.

### 2.5. Statistical Analysis

The two-tailed Wilcoxon rank-sum test was performed to estimate the statistical differences of combined index between early and late recurrence. To assess the predictive effect, a receiver operating characteristic curve analysis was used to estimate the area under the curve. Disease-free curves were calculated using the Kaplan–Meier method, and distributions were compared using the log-rank test. Disease-specific overall survival was calculated from the date of diagnosis until disease-caused death or the end of follow-up. A univariate COX regression analysis was used to assess the association between each variable and survival/recurrence. A Cox proportional hazards model was used in the multivariate analyses and was also used to estimate Hazard Ratios (HRs) and their 95% confidence intervals (CIs).

## 3. Results

### 3.1. Patient Enrollment and Characteristics

**Figure A1:**
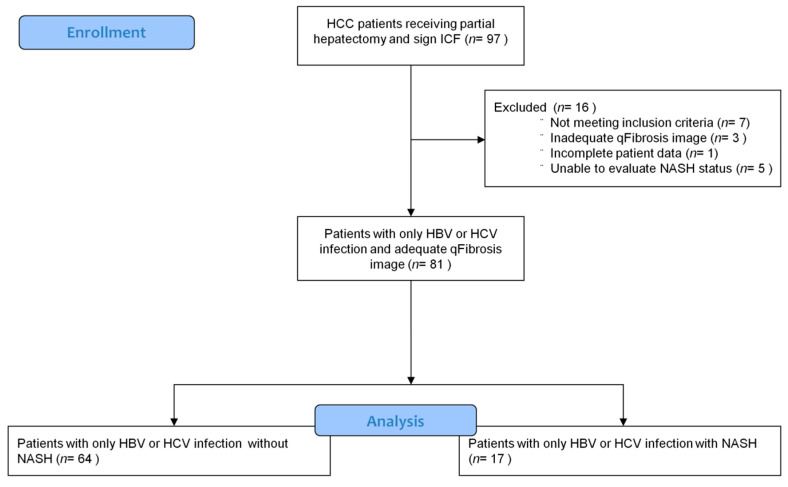
Flowchart for patient enrollment (ICF: Informed Consent Form).

A total of 97 patients who had received curative hepatectomy for HCC from June 2007 to January 2013 at National Cheng Kung University Hospital and Chi Mei Medical Center were screened. Among 97 patients, 81 patients were finally enrolled, and 16 patients were excluded due to co-infection of HBV and HCV, incomplete patient data, inadequate qFibrosis image or inevaluable NASH status. These 81 patients were further separated into 2 groups, 64 patients without NASH and 17 patients with NASH features (Figure A1). The characteristics of the 81 patients studied are summarized in Table A1. 

Most of the enrolled patients in this study were treatment naïve. In the non-NASH group, local treatment such as transarterial embolization (TAE), radiofrequency ablation (RFA) or partial hepatectomy were performed previously in 5 patients; TAE and RFA were done in 2 patients in the NASH group. No patients received systemic treatment before enrollment. After recurrence, 16 ptients received RFA, 16 patients received transcatheter arterial chemoembolization (TACE)/TAE, 10 patients had medical treatment, 8 patients received surgical intervention, 8 patients had radiotherapy, 2 patients received hepatic arterial infusion chemotherapy, and 2 patients received percutaneous ethanol injection.

### 3.2. Features for Constructing the Combined Index

qFibrosis is a powerful automated computer-aided image system intended to assess patterns of collagen and quantify liver fibrosis. We used this new technology to evaluate the fibrotic status of hepatic tissue removed from the enrolled patients. To predict early recurrence of viral infection related to HCC after a hepatectomy, a “combined index” was calculated using qFibrosis with the tissue sample for the 64 non-NASH patients. The acquired SHG/TPEF images of liver sections were processed and the combined index was obtained. 

The model construction process selected 18 features to construct the prediction model and compute the combined index. Of the 18 features, 9 were in the 100 features and other 9 features were the relativistic features. The coefficients of features for the linear model were estimated based on the 64 samples (Table 1). 

### 3.3. Using the Combined Index of qFibrosis to Predict Early Recurrence of HCC

**Figure 3:**
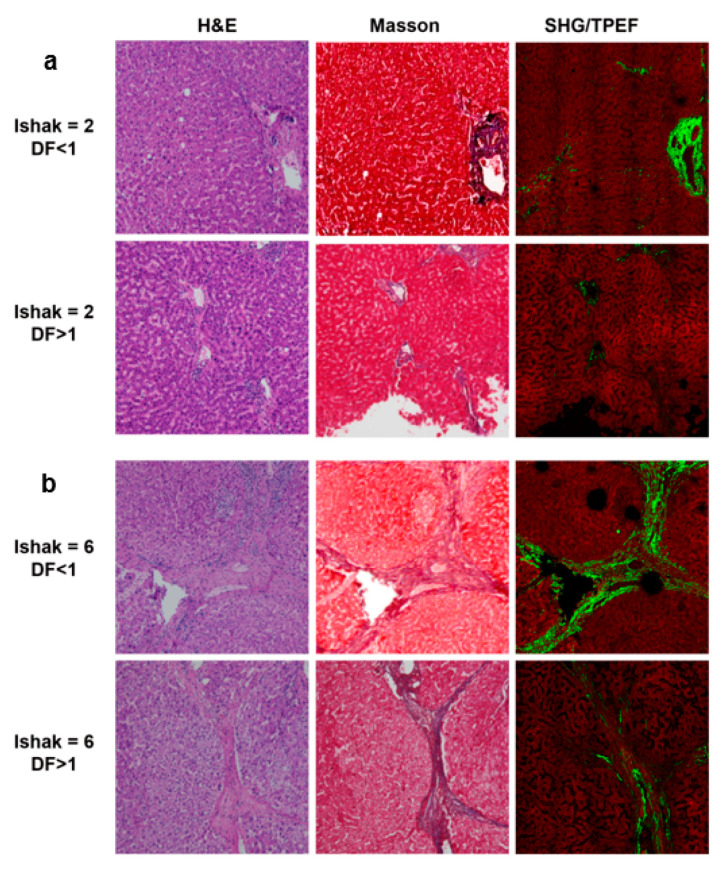
H&E staining, Masson staining, and SHG/TPEF images in the HCC liver samples. (**a**) Ishak score = 2, disease free (DF) < 1 year and > 1 year. (**b**) Ishak score = 6, DF <1 year and >1 year.

**Figure 4:**
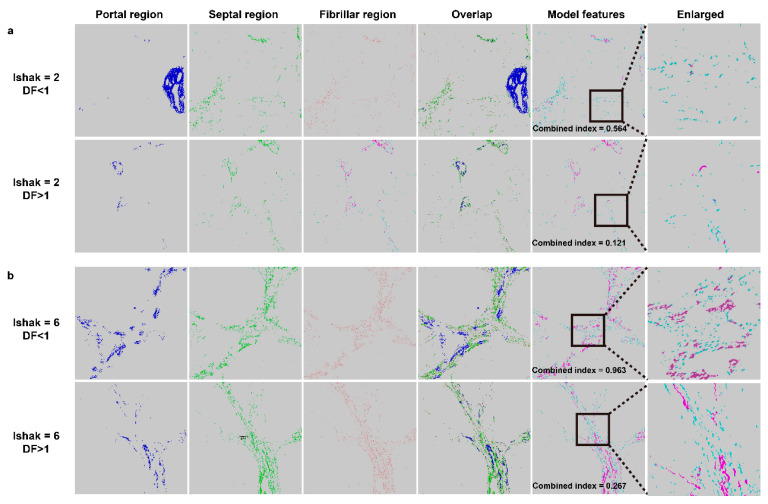
Examples of different collagen regions. (**a**) Ishak score = 2, disease free (DF) < 1 year and > 1 year. (**b**) Ishak score = 6, DF < 1 year and > 1 year. Overlap region includes three collagen patterns (portal/septal/fibrillar). Model features shows two collagen features including aggregated (purple color) and distributed (blue-green color) collagen in septal region used was for the combined index, which is to predict early recurrence in patients with hepatocellular carcinoma after curative hepatectomy.

**Figure 5:**
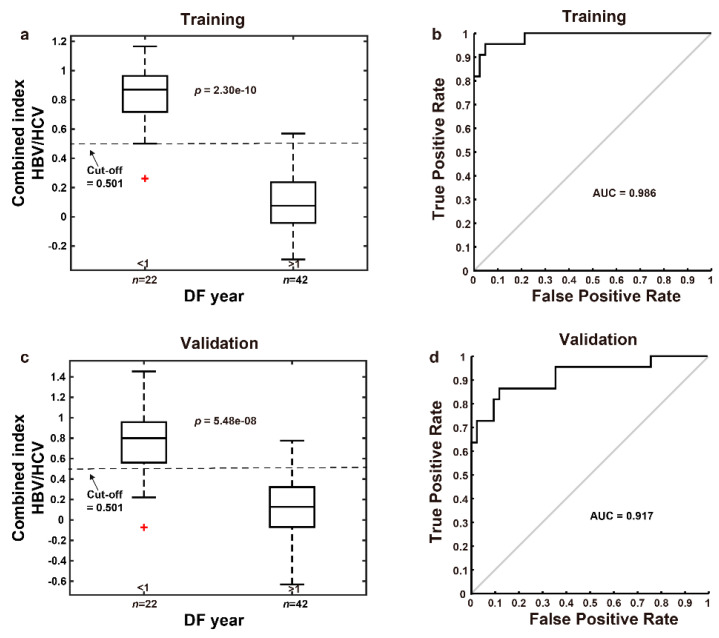
ROC curves for the prediction of early recurrence versus late recurrence for the HBV or HCV patients without NASH. (**a**) A combined index cut-off value of 0.501 is capable of differentiating between early and late recurrence in the training group. (**b**) ROC curve for combined index showed great predictive value of early recurrence (AUC = 0.986) in the training group. (**c**) The predicted combined index values for 64 patients were calculated by leave-one-out cross-validation method. (**d**) ROC curve for the combined index predicted by leave-one-out cross-validation method showed great predictive value of early recurrence (AUC = 0.917). Note: The red plus sign represents outlier.

We employed qFibrosis to evaluate the fibrotic status of the hepatic tissue from patients with HCC, and the architectural features of the studied collagen were separated into 3 regions: portal, septal, and fibrillar, as illustrated in Figure 1a. In Figure 3, we show the results of hematoxylin and eosin (H&E) staining, Masson staining, and SHG/TPEF images in the HCC liver samples with early and late recurrence. The detailed different collagen regions and part of the modal features used for the combined index were shown in Figure 4. Although the Ishak scale scores were the same (Ishak both = 2 in Figure 4a; both = 6 in Figure 4b), the combined index can be used to tell the difference in the fibrotic status, which may predict early and late recurrence (combined index = 0.564 and 0.121 in Figure 4a; = 0.963 and 0.267 in Figure 4b). These results indicate that the combined index was better able to distinguish the fibrotic status compared to the conventional Ishak scale. From the training data, we found that a combined index cut-off value of 0.501 was useful to differentiate early recurrence (<1 year; combined index > 0.501) from late or no recurrence (≥1 year; combined index ≤ 0.501) (Figure 5a), where the receiver operator characteristic (ROC) curves for the prediction of early recurrence versus late or no recurrence was 0.986 (AUC = 0.986, Figure 5b). The validation confirmed that the combined index showed high performance (AUC = 0.917, Figure 5c,d).

**Figure A2:**
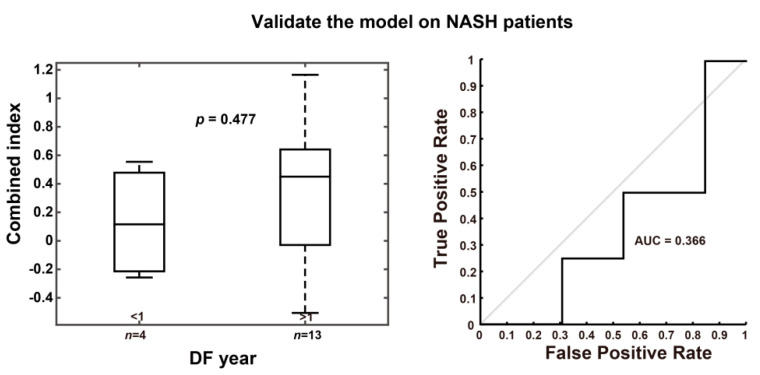
ROC curves for the prediction of early recurrence versus late recurrence for NASH patients.

**Figure A3:**
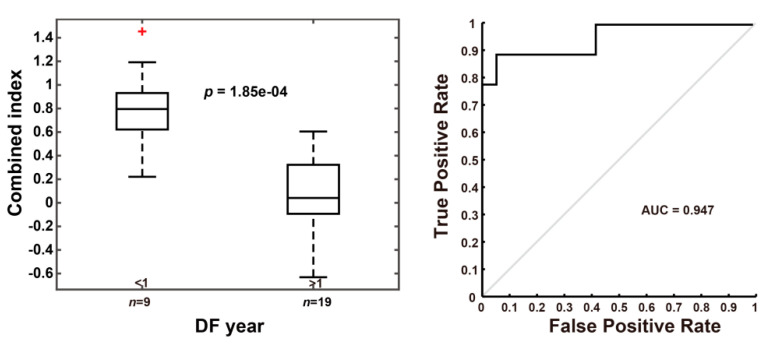
ROC curves for the prediction of early recurrence versus late recurrence for cirrhosis patients. Note: The red plus sign represents outlier.

We also applied the combined index in other 17 patients having HCC with NASH features, and the result suggested that it is a poor predictor for early recurrence in these NASH patients (AUC = 0.336, Table A2 and Figure A2). On the other hand, the current model showed promising performance in the 28 cirrhotic patients (AUC = 0.947, Figure A3).

#### 3.3.1. Combined Index Is a Better Predictor of Early Recurrence than Alpha Fetoprotein

**Figure 6:**
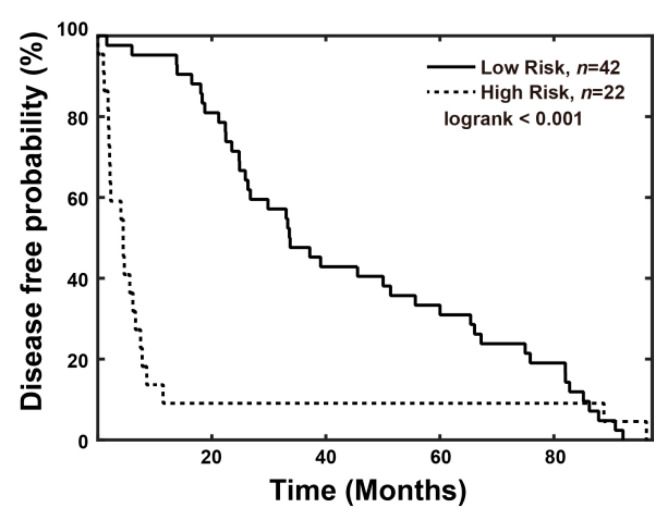
Disease-free probability analysis for HCC patients. A significant difference was noted between the high-risk group (combined-index > 0.501) and low risk group (combined-index ≤0.501) (*n* = 22 and 42, *p* < 0.001).

**Figure A4:**
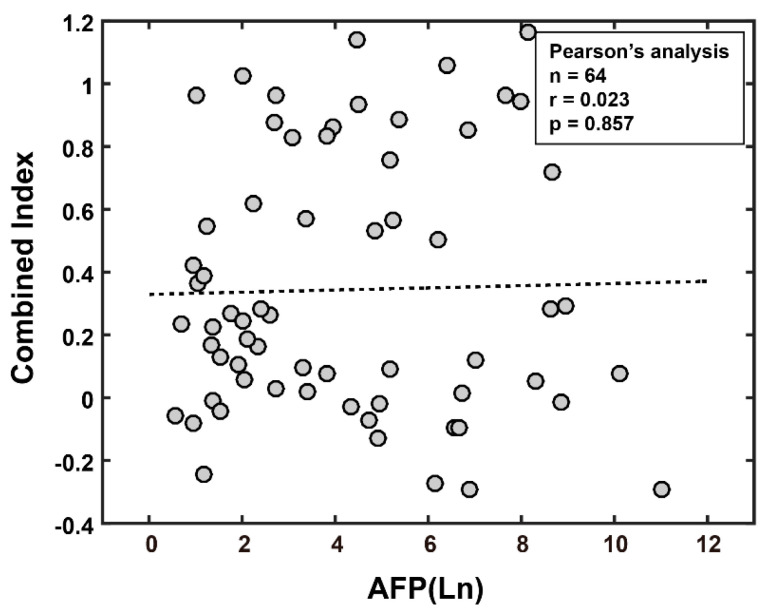
Correlation of AFP level and combined index. In patients after hepatectomy, there is poor correlation shown between the AFP level and combined index as determined with a Pearson’s analysis.

Previous studies reported that HCC patients with high-level serum AFP (>20 ng/mL) had higher postoperative 2-year recurrence rates and lower 24-month survival rates [18,19]. Compared to elevated alpha fetoprotein (AFP >20 ng/mL), the high combined index (>0.501) showed better predictive value for early recurrence, including AUC (0.917 vs. 0.595), sensitivity (81.8% vs. 68.2), specificity (90.5% vs. 47.6%), false positive rate (9.5% vs. 52.4%), and false negative rate (18.2% vs. 31.8%), as shown in Table A3. Disease-free probability was lower in the high-risk group (combined index >0.501, *n* = 22) than in the low-risk group (combined index ≤ 0.501, *n* = 42), and the p value was 0.035 (Figure 6). In addition, the correlation of the AFP level and combined index was low in patients after hepatectomy using a Pearson’s analysis (Figure A4).

In the univariate analysis, vascular invasion (yes vs. no, *p* = 0.021), tumor size (>5 vs. ≤5 cm, *p* = 0.005), pathological stage (III/IV vs. I/II, *p* = 0.029), clinical stage (III/IV vs. I/II, *p* = 0.005), and the combined index (>0.501 vs. ≤0.501, *p* < 0.001) seemed to predict poor survival (Table 2).

Using a Cox proportional hazards analysis, we found the combined index (high risk vs. low risk; HR: 3.821, 95% C.I.: 1.596–9.153, *p* = 0.003) and Model for End-Stage Liver Disease (MELD) score (≥10 vs. ≤9; HR: 4.167, 95% C.I.: 1.173–14.803, *p* = 0.027) to be poor prognostic factors of disease-free survival. We also found the combined index (high risk vs. low risk; HR: 4.509, 95% C.I.: 1.366–12.058, *p* = 0.012), AFP (>20 vs. ≤ 20; HR: 4.639, 95% C.I.: 1.358–15.84, *p* = 0.014), MELD score (≥10 vs. ≤9; HR: 7.628, 95% C.I.: 1.393–41.757, *p* = 0.019) and Clinical Stage (III/IV vs. I/II; HR: 4.285, 95% C.I.: 1.160–15.825, *p* = 0.029) to be poor prognostic factors of overall survival (Table 3). According to these data, we conclude that the combined index has better predictive value of early recurrence as compared to the AFP.

#### 3.3.2. The Combined Index Significantly Predicts Early Recurrence as Compared to Other Regions and Features of Fibrosis

**Figure 7:**
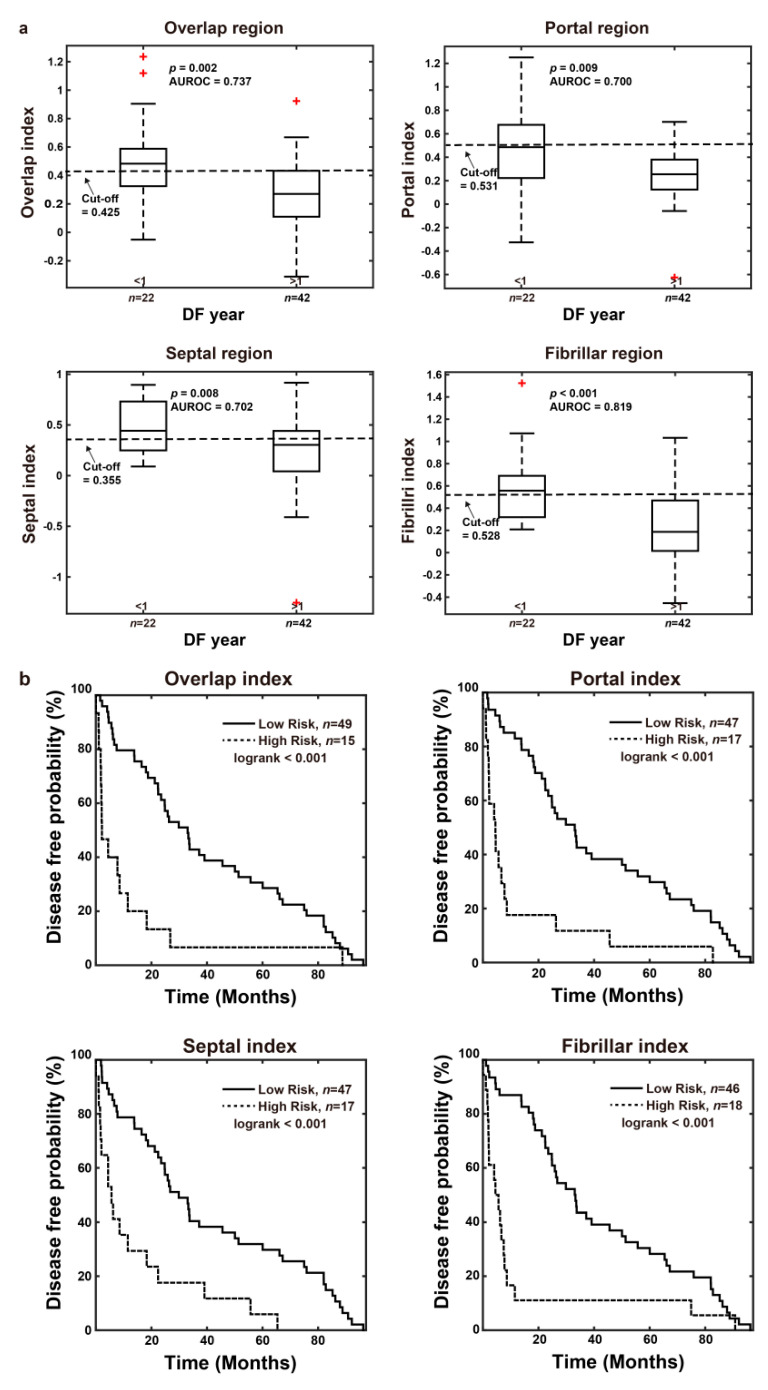
The prediction of early recurrence using features in the overlap, portal, septal, and fibrillar regions by leave-one-out cross-validation method. The features of non-tumor liver in these regions show poorer predictive ability compared with the combined index. (**a**) Box plots. The cut off values were determined by the training data (*n* = 64). (**b**) Disease-free probability analysis. The high-risk group and low risk group were separated by the corresponding cut-off value. Note: The red plus sign represents outlier.

To further investigate the correlation with early recurrence, we evaluated fibrotic features in different regions of non-tumor hepatic tissue using qFibrosis. In overlap, portal, septal, and fibrillar regions, 8, 11, 11, and 13 features were selected, respectively (Tables S2–S5). The results of the leave-one-out cross-validation method showed higher correlations in the fibrillar region (AUC = 0.819) than in other (AUC = 0.700 in portal; 0.702 in septal) or overlap regions (AUC = 0.737) (Figure 7a). However, the combined index still exhibited the best correlation (AUC = 0.917). The disease-free probability according to high and low risk by fibrotic features in the overlap, portal, septal, and fibrillar regions of a non-tumor liver are shown in Figure 7b (*p* < 0.001 in four regions). The features of the diagnosis of early recurrence are listed in Table S6. With these data, the combined index is suggested to be most predictive for early recurrence as compared to other regions.

## 4. Discussion

Currently, HCC is still one of the leading causes of cancer deaths worldwide. Partial hepatectomy remains the most commonly used method to cure patients. However, high recurrence rates have been observed after curative intent hepatectomy. According to previous studies, liver fibrosis increases the risk of intrahepatic recurrence after hepatectomy or radiofrequency ablation for HCC [7,20]. Traditional histological fibrotic staging systems, such as the Ishak fibrotic score, although the current standard, are criticized for their subjective interpretation due to either sampling error or observer variations. qFibrosis provides a fully-quantitative method incorporating histological features to obtain more accurate fibrosis scoring for the liver. Our study results indicated that the combined index calculated using qFibrosis may predict early recurrence of HCC after curative intent hepatectomy.

qFibrosis has shown its ability to perform accurate fibrotic scoring of hepatic tissue in animal models and chronic hepatitis B patients. Besides, it had been established as a better way for screening and enrollment of NASH patients in clinical trials [21,22]. Our results using the recurrence prediction model in 64 HCC patients after hepatectomy indicated that early recurrence can be predicted when the combined index is more than 0.501. Ko et al. reported that histological evidence of fibrosis of the underlying liver tissue is the most significant predictive factor of intrahepatic recurrence. Our novel method can be used to determine differences in fibrotic status when the samples are scored the same by the Ishak system, as shown in Figure 4a,b. Therefore, using this method will make it possible to follow high-risk patients more carefully and also consider other treatment according to the risks of disease recurrence.

It is known that there are also many non-invasive tools for evaluation of fibrotic status [23,24,25,26,27]. Many of them use serum markers, which may be influenced largely by the inflammation status of the patient. In addition, some of these markers may not be specific for the liver. Some image-based non-invasive methods arrive at indeterminate results for fibrotic status in up to 33% of cases, which is not satisfactory by today’s medical standards [28]. Artificial intelligence has been widely applied in modern precision medicine for several years, with some applications focusing on digital pathology images [29], others on interpretation of multiple data or radiological images [30,31,32,33]. In our study, we simply used qFibrosis to obtain the accurate fibrotic status of the resected liver sample, and processed the specific features with the clinical data using multi-dimensional artificial intelligence analysis. As the result, the “combined index” showed good prediction ability in early recurrence of HCC.

Viral and non-viral related HCC are thought to have different pathologic mechanisms in progression of normal liver tissue to liver cancer. We had applied the combined index, which derived from the viral related non-NASH HCC patients, in other HCC patients with NASH features, and it was unable to predict early recurrence in these NASH patients. Therefore, the fibrotic pattern of liver tissue may be different in the viral and non-viral related HCC patients, and further study is needed.

There were some limitations to this study. First, it was hard for us to collect another group of patients for external validation, so we used a leave-one-out cross-validation method to overcome this problem. Second, although qFibrosis can provide more accurate fibrotic status than conventional histological methods, sampling error may still have some influence on the qFibrosis score. Besides, our study was unable to provide competing risk analysis as Metroticket 2.0 model used in liver transplantation patients owing to the complicated clinical situations and the study design [34,35]. Finally, our method needs liver tissue to obtain its qFibrosis score, so it is not a non-invasive assessment of liver fibrosis.

## 5. Conclusions

This is the first study using the combined index calculated with qFibrosis to allow accurate quantification of fibrotic status of the peri-tumor liver tissue, and it also provides a good tool for prediction of early hepatocellular carcinoma recurrence after curative intent surgery. Clinically, delayed treatment for recurrent disease may decrease the patient’s life expectancy. As a result, patients identified to be at high-risk by the combined index should be monitored in shorter time intervals, and further intervention may be provided earlier if needed.

## Figures and Tables

**Table 1:** The list of estimated coefficients of 18 selected features for constructing the combined index of the HBV or HCV patients without NASH.

No.	Features	Estimated Coefficients	Region
0	Intercept	3.838	-
1	SHG	4.300	Overlap
2	StrOrientation	1.280	Overlap
3	StrAreaPA	−2.413	Portal
4	StrAreaPD	−1.269	Portal
5	NoThickStrS	3.182	Septal
6	NoThinStrSA	−2.486	Septal
7	Fibrillar	−2.591	Fibrillar
8	NoThickStrF	−1.889	Fibrillar
9	NoThickStrFA	1.735	Fibrillar
10	NoShortStr/NoLongStr	−3.733	Overlap/Overlap
11	StrLengthP/StrWidthP	−0.859	Portal/Portal
12	NoThickStrPD/NoStrPD	−0.771	Portal/Portal
13	NoThinStrPD/NoThickStrPD	−0.599	Portal/Portal
14	SeptalAGG/Septal	−1.782	Septal/Septal
15	StrLengthSD/StrWidthSD	−0.761	Fibrillar/Fibrillar
16	NoThinStrFA/NoThickStrFA	0.957	Fibrillar/Fibrillar
17	NoThickStrFD/NoStrFD	−0.443	Fibrillar/Fibrillar
18	StrLengthFD/StrWidthFD	1.067	Fibrillar/Fibrillar

**Table 2:** Univariate Analysis of Variables Potentially Predictive of Survival in HCC.

Variable	Number of Patients	Death		*p*-Value
		Number	Percent	
Gender				0.126
Male	47	21	44.7	
Female	17	4	23.5	
Groups				0.703
HBV + cirrhosis	20	7	35.0	
HBV	31	13	41.9	
HCV+cirrhosis	8	4	50.0	
HCV	5	1	20.0	
Liver cirrhosis				0.974
No	36	14	38.9	
Yes	28	11	39.3	
Histologic grade				0.431
Well	6	1	16.7	
Moderate	50	20	40.0	
Poor	8	4	50.0	
Vascular invasion				0.021 *
No	37	10	27.0	
Yes	27	15	55.6	
Tumor size (cm)				0.005 *
≤5	37	9	24.3	
> 5	27	16	59.3	
AFP (ng/mL)				0.066
≤20	27	7	25.9	
>20	37	18	48.6	
Pathological Stage				0.029 *
Stage I, II	54	18	33.3	
Stage III, IV	10	7	70.0	
Clinical Stage				0.005 *
Stage I, II	50	15	30.0	
Stage III, IV	14	10	71.4	
Combined Index				<0.001 *
≤0.501	42	9	21.4	
>0.501	22	16	72.7	

* *p* < 0.05.

**Table 3:** Cox Proportional Hazards Analysis of Prognostic Parameters in HCC.

Factors	Multivariate			
	HR	95%CI		*p*-Value
Disease-free survival				
Gender (Female vs. Male)	0.804	0.352	1.838	0.605
Liver cirrhosis (Yes vs. No)	1.344	0.660	2.735	0.415
Histologic grade (Moderate vs. Well)	0.815	0.260	2.554	0.725
Histologic grade (Poor vs. Well)	1.361	0.324	5.709	0.674
Vascular invasion (Yes vs. No)	0.635	0.265	1.518	0.307
Tumor size (>5 vs. ≤5)	1.546	0.688	3.476	0.291
AFP (>20 vs. ≤20)	1.510	0.651	3.501	0.337
Pathological Stage (III/IV vs. I/II)	1.960	0.692	5.547	0.205
Clinical Stage (III/IV vs. I/II)	2.029	0.816	5.047	0.128
MELD score (≥10 vs. ≤9)	4.167	1.173	14.803	0.027 *
BCLC Stage (B/C vs. 0/A)	1.444	0.601	3.466	0.411
Combined Index (High risk vs. Low risk)	3.821	1.596	9.153	0.003 *
Overall survival				
Gender (Female vs. Male)	0.330	0.092	1.176	0.087
Liver cirrhosis (Yes vs. No)	1.517	0.580	3.970	0.395
Histologic grade (Moderate vs. Well)	1.998	0.168	23.790	0.584
Histologic grade (Poor vs. Well )	3.534	0.218	57.208	0.374
Vascular invasion (Yes vs. No)	2.137	0.728	6.277	0.167
Tumor size (>5 vs. ≤5)	1.467	0.470	4.584	0.509
AFP (>20 vs. ≤20)	4.639	1.358	15.840	0.014 *
Pathological Stage (III/IV vs. I/II)	0.396	0.110	1.425	0.156
Clinical Stage (III/IV vs. I/II)	4.285	1.160	15.825	0.029 *
MELD score (≥10 vs. ≤9)	7.628	1.393	41.757	0.019 *
BCLC Stage (B/C vs. 0/A)	1.061	0.367	3.069	0.913
Combined Index (High risk vs. Low risk)	4.059	1.366	12.058	0.012 *

* *p* < 0.05.

## Data Availability

The data presented in this study are available in the article and supplementary materials.

## Supplementary Materials

The following are available online at https://www.mdpi.com/article/10.3390/cancers13215323/s1, Table S1: The inclusion and exclusion criteria, Table S2: The list of estimated coefficients of 8 selected features in the overlap region, Table S3: The list of estimated coefficients of 11 selected features in the portal region, Table S4: The list of estimated coefficients of 11 selected features in the septal region, Table S5: The list of estimated coefficients of 13 selected features in the fibrillar region, Table S6: P values of 100 features for the diagnosis of early recurrence.

## Appendix A

**Table A1:** Demographic and disease characteristics of the enrolled patients.

Characteristics	NASH Patients (*n* = 64)	Non-NASH Patients (*n* = 17)	Total (*n* = 81)
Age (years)			
median (range)	55 (27–81)	60 (33–74)	56 (27–81)
Gender			
Male	47 (73%)	10 (59%)	57 (70%)
Female	17 (27%)	7 (41%)	24 (30%)
Viral hepatitis type			
HBV	51 (80%)	13 (76%)	64 (79%)
HCV	13 (20%)	4 (24%)	17 (21%)
Liver cirrhosis			
Yes	28 (44%)	11 (65%)	39 (48%)
No	36 (56%)	6 (35%)	42 (52%)
Histologic grade			
Well	6 (9%)	2 (12%)	8 (10%)
Moderate	50 (78%)	14 (82%)	64 (79%)
Poor	8 (13%)	1 (6%)	9 (11%)
Vascular invasion			
Yes	27 (42%)	2 (12%)	29 (36%)
No	37 (58%)	15 (88%)	52 (64%)
Tumor size (cm)			
≤5	37 (58%)	16 (94%)	53 (65%)
>5	27 (42%)	1 (6%)	28 (35%)
AFP (ng/mL)			
≤20	27 (42%)	10 (59%)	37 (46%)
>20	37 (58%)	7 (41%)	44 (54%)
Pathological Stage			
I/II	54 (84%)	17 (100%)	71 (88%)
III/IV	10 (16%)	0 (0%)	10 (12%)
Clinical Stage			
I/II	50 (78%)	16 (94%)	66 (81%)
III/IV	14 (22%)	1 (6%)	15 (19%)
MELD score			
≤9	59 (92%)	16 (94%)	75 (93%)
≥10	5 (8%)	1 (6%)	6 (7%)
BCLC Stage			
0/A	50 (78%)	14 (82%)	64 (79%)
B/C	14 (22%)	3 (18%)	17 (21%)
Child-Pugh class			
A5	62 (97%)	17 (100%)	79 (98%)
A6	2 (3%)	0 (0%)	2 (2%)
ECOG PS			
0	61 (95%)	16 (94%)	77 (95%)
1	3 (5%)	1 (6%)	4 (5%)
Creatinine level (mg/dL)			
Median (IQR)	0.9 (0.79–1.0)	0.8 (0.665–0.94)	0.9 (0.705–0.995)
Liver function enzyme			
AST (mg/dL)			
Median (IQR)	48.5 (33–73)	37 (29–67.5)	47 (33–71)
ALT (mg/dL)			
Median (IQR)	41.5 (31.5–80.25)	33.5 (28.5–37.75)	37.5 (30–64.5)
Comorbidities			
Hypertension	13 (20%)	8 (47%)	21 (26%)
Diabetes mellitus	5 (8%)	3 (18%)	8 (10%)
Chronic kidney disease	4 (6%)	1 (6%)	5 (6%)
Coronary artery disease	1 (2%)	0 (0%)	1 (1%)
Cerebrovascular accident	1 (2%)	0 (0%)	1 (1%)

Abbreviation: AFP: alpha-fetoprotein; MELD score: Model for End-Stage Liver Disease score; BCLC stage: Barcelona Clinic Liver Cancer stage; ECOG PS: Eastern Cooperative Oncology Group performance status; IQR: interquartile range; AST: aspartate aminotransferase; ALT: alanine aminotransferase.

## Appendix B

**Table A2:** Validation of the model on NASH patients.

Group	*p* Value between DF Year < 1 and > 1	AUROC
NASH patients (*n* = 17)	0.477	0.366

## Appendix C

**Table A3:** AUC: sensitivity, specificity, false positive rate (FPR), and false negative rate (FNR) for the combined index and AFP for the prediction of early recurrence.

Characteristics	Combined Index > 0.501	AFP > 20 ng/mL
AUC	0.917	0.595
Sensitivity, %	81.8	68.2
Specificity, %	90.5	47.6
FPR, %	9.5	52.4
FNR, %	18.2	31.8
