# Supplementary material for: Predict Early Recurrence of Resectable Hepatocellular Carcinoma Using Multi-Dimensional Artificial Intelligence Analysis of Liver Fibrosis
Source: Cancers (Basel). 2021 Oct 23;13(21):5323. doi: 10.3390/cancers13215323 (PMC8582529; doi:10.3390/cancers13215323)
Supplement: Supplementary file 1 [file cancers-13-05323-s001.zip › cancers-1425671-supplementary.pdf]

Table S1: The inclusion and exclusion criteria.

Inclusion criteria
1. Signed informed consent form.
2. Age ≧ 20 years at time of signing informed consent form.
3. Diagnosed as resectable HCC, and curative intent surgical resection is planned.
4. Patient with known HBV or HCV infection.
5. Life expectancy ≧ 3 months.
**Exclusion criteria**
1. Patient with co-infection of HBV and HCV.
2. Inadequate tissue samples after surgical management, including tumor tissue and non-tumor liver tissue.
3. History of other malignancy within 2 years prior to screening, with the exception of malignancies with a negligible risk of metastasis or death, such as treated carcinoma in situ of the cervix, non-melanoma skin carcinoma, localized prostate cancer, ductal carcinoma in situ or Stage I uterine cancer.

Table S2: The list of estimated coefficients of 8 selected features in the overlap region.

No.	Features	Estimated Coefficients
**0**	Intercept	-0.272
**1**	SHG	2.658
**2**	Dis	-1.551
**3**	StrOrientation	1.060
**4**	NoXlink	-1.469
**5**	Dis/SHG	1.109
**6**	NoShortStr/NoStr	5.272
**7**	NoShortStr/NoLongStr	-8.664
**8**	StrLength/StrWidth	-2.199

Table S3: The list of estimated coefficients of 11 selected features in the portal region.

No.	Features	Estimated Coefficients
**0**	Intercept	2.130
**1**	NoShortStrP	-3.751
**2**	StrWidthP	2.928
**3**	NoThickStrPA	1.179

**4**	PortalDIS/PortalAGG	1.345
**5**	NoShortStrPA/NoStrPA	0.712
**6**	NoThinStrPA/NoStrPA	-3.791
**7**	NoThinStrPA/NoThickStrPA	4.402
**8**	StrLengthPA/StrWidthPA	-1.762
**9**	NoThickStrPD/NoStrPD	-0.979
**10**	NoShortStrPD/NoLongStrPD	-0.452
**11**	NoThinStrPD/NoThickStrPD	-0.945

Table S4: The list of estimated coefficients of 11 selected features in the septal region.

No.	Features	Estimated Coefficients
**0**	Intercept	4.439
**1**	NoShortStrS	-0.277
**2**	NoThickStrSD	3.846
**3**	StrAreaSD	-2.823
**4**	SeptalAGG/Septal	-3.732
**5**	SeptalDIS/SeptalAGG	-2.628
**6**	NoShortStrSA/NoStrSA	-4.608
**7**	NoShortStrSA/NoLongStrSA	1.607
**8**	StrLengthSA/StrWidthSA	-2.001
**9**	NoShortStrSD/NoLongStrSD	-0.646
**10**	NoThinStrSD/NoThickStrSD	-1.199
**11**	StrLengthSD/StrWidthSD	3.715

Table S5: The list of estimated coefficients of 13 selected features in the fivrillar region.

No.	Features	Estimated Coefficients
**0**	Intercept	-0.199
**1**	Fibrillar	-2.736
**2**	FibrillarAGG	2.731
**3**	NoThickStrF	-0.778
**4**	NoThickStrFA	1.633
**5**	NoThinStrFD	5.302
**6**	StrLengthFD	-9.733
**7**	StrWidthFD	4.515
**8**	FibrillarDIS/FibrillarAGG	0.950
**9**	NoThinStrFA/NoThickStrFA	0.659
**10**	NoThinStrFD/NoStrFD	-0.647
**11**	NoThickStrFD/NoStrFD	-0.731

**12**	NoThinStrFD/NoThickStrFD	0.824
**13**	StrLengthFD/StrWidthFD	1.769

Table S6: P values of 100 features for the diagnosis of early recurrence. * Statistical significance level was set as p<0.05.

Regions	*Features*	Description	P value
**Overlap N = 16**	*SHG*	*Percentage of total collagen in overlap region*	0.022*
	Agg	*Percentage of aggregated collagen in overlap region*	0.045*
	Dis	*Percentage of distributed collagen in overlap region*	0.007*
	NoStr	*Number of strings in overlap region*	0.014*
	NoShortStr	*Number of short strings in overlap region*	0.021*
	NoLongStr	*Number of long strings in overlap region*	0.011*
	NoThinStr	*Number of thin strings in overlap region*	0.035*
	NoThickStr	*Number of thick strings in overlap region*	0.014*
	StrArea	*Total area of all strings in overlap region*	0.045*
	StrLength	*Total length of all strings in overlap region*	0.012*
	StrWidth	*Total width of all strings in overlap region*	0.015*
	StrEccentricity	*Total eccentricity of all strings in overlap region*	0.017*
	StrSolidity	*Total solidity of all strings in overlap region*	0.017*
	StrPerimeter	*Total perimeter of all strings in overlap region*	0.018*
	StrOrientation	*Total orientation of all strings in overlap region*	0.000*
	NoXlink	*Number of cross-links of strings in overlap region*	0.029*
Count (p<0.05)			*16*
**Portal N = 28**	*Portal*	*Percentage of total collagen in portal region*	0.196
	PortalAGG	*Percentage of aggregated collagen in portal region*	0.206
	PortalDIS	*Percentage of distributed collagen in portal region*	0.377
	NoStrP	*Number of strings in portal region*	0.074
	NoShortStrP	*Number of short strings in portal region*	0.076
	NoLongStrP	*Number of long strings in portal region*	0.069
	NoThinStrP	*Number of thin strings in portal region*	0.109
	NoThickStrP	*Number of thick strings in portal region*	0.086
	StrAreaP	*Total area of all strings in portal region*	0.244
	StrLengthP	*Total length of all strings in portal region*	0.140
	StrWidthP	*Total width of all strings in portal region*	0.112
	NoStrPA	*Number of aggregated strings in portal region*	0.076
	NoShortStrPA	*Number of short and aggregated in portal region*	0.055
	NoLongStrPA	*Number of long and aggregated in portal region*	0.106
	NoThinStrPA	*Number of thin and aggregated in portal region*	0.168

	NoThickStrPA	*Number of thick and aggregated in portal region*	0.083
	StrAreaPA	*Total area of aggregated in portal region*	0.326
	StrLengthPA	*Total length of aggregated in portal region*	0.196
	StrWidthPA	*Total width of aggregated in portal region*	0.109
	NoStrPD	*Number of distributed strings in portal region*	0.369
	NoShortStrPD	*Number of short and distributed in portal region*	0.392
	NoLongStrPD	*Number of long and distributed in portal region*	0.153
	NoThinStrPD	*Number of thin and distributed in portal region*	0.312
	NoThickStrPD	*Number of thick and distributed in portal region*	0.355
	StrAreaPD	*Total area of distributed in portal region*	0.377
	StrLengthPD	*Total length of distributed in portal region*	0.369
	StrWidthPD	*Total width of distributed in portal region*	0.385
	NoXlinkP	*Number of cross-links of all strings in portal region*	0.115
Count (p<0.05)			*0*
**Septal N = 28**	*Septal*	*Percentage of total collagen in septal region*	0.065
	SeptalAGG	*Percentage of aggregated collagen in septal region*	0.122
	SeptalDIS	*Percentage of distributed collagen in septal region*	0.014*
	NoStrS	*Number of strings in septal region*	0.013*
	NoShortStrS	*Number of short strings in septal region*	0.014*
	NoLongStrS	*Number of long strings in septal region*	0.017*
	NoThinStrS	*Number of thin strings in septal region*	0.037*
	NoThickStrS	*Number of thick strings in septal region*	0.013*
	StrAreaS	*Total area of all strings in septal region*	0.091
	StrLengthS	*Total length of all strings in septal region*	0.022*
	StrWidthS	*Total width of all strings in septal region*	0.017*
	NoStrSA	*Number of aggregated strings in septal region*	0.045*
	NoShortStrSA	*Number of short and aggregated in septal region*	0.059
	NoLongStrSA	*Number of long and aggregated in septal region*	0.029*
	NoThinStrSA	*Number of thin and aggregated in septal region*	0.155
	NoThickStrSA	*Number of thick and aggregated in septal region*	0.028*
	StrAreaSA	*Total area of aggregated in septal region*	0.168
	StrLengthSA	*Total length of aggregated in septal region*	0.041*
	StrWidthSA	*Total width of aggregated in septal region*	0.032*
	NoStrSD	*Number of distributed strings in septal region*	0.022*
	NoShortStrSD	*Number of short and distributed in septal region*	0.018*
	NoLongStrSD	*Number of long and distributed in septal region*	0.044*
	NoThinStrSD	*Number of thin and distributed in septal region*	0.025*

	NoThickStrSD	*Number of thick and distributed in septal region*	0.016*
	StrAreaSD	*Total area of distributed in septal region*	0.029*
	StrLengthSD	*Total length of distributed in septal region*	0.035*
	StrWidthSD	*Total width of distributed in septal region*	0.017*
	NoXlinkS	*Number of cross-links of all strings in septal region*	0.040*
Count (p<0.05)			*22*
**Fibrillar N = 28**	*Fibrillar*	*Percentage of total collagen in fibrillar region*	0.030*
	FibrillarAGG	*Percentage of aggregated collagen in fibrillar region*	0.086
	FibrillarDIS	*Percentage of distributed collagen in fibrillar region*	0.015*
	NoStrF	*Number of strings in fibrillar region*	0.017*
	NoShortStrF	*Number of short strings in fibrillar region*	0.424
	NoLongStrF	*Number of long strings in fibrillar region*	0.018*
	NoThinStrF	*Number of thin strings in fibrillar region*	0.013*
	NoThickStrF	*Number of thick strings in fibrillar region*	0.083
	StrAreaF	*Total area of all strings in fibrillar region*	0.016*
	StrLengthF	*Total length of all strings in fibrillar region*	0.016*
	StrWidthF	*Total width of all strings in fibrillar region*	0.015*
	NoStrFA	*Number of aggregated strings in fibrillar region*	0.019*
	NoShortStrFA	*Number of short and aggregated in fibrillar region*	0.661
	NoLongStrFA	*Number of long and aggregated in fibrillar region*	0.019*
	NoThinStrFA	*Number of thin and aggregated in fibrillar region*	0.029*
	NoThickStrFA	*Number of thick and aggregated in fibrillar region*	0.012*
	StrAreaFA	*Total area of aggregated in fibrillar region*	0.023*
	StrLengthFA	*Total length of aggregated in fibrillar region*	0.025*
	StrWidthFA	*Total width of aggregated in fibrillar region*	0.021*
	NoStrFD	*Number of distributed strings in fibrillar region*	0.020*
	NoShortStrFD	*Number of short and distributed in fibrillar region*	0.313
	NoLongStrFD	*Number of long and distributed in fibrillar region*	0.019*
	NoThinStrFD	*Number of thin and distributed in fibrillar region*	0.020*
	NoThickStrFD	*Number of thick and distributed in fibrillar region*	0.174
	StrAreaFD	*Total area of distributed in fibrillar region*	0.025*
	StrLengthFD	*Total length of distributed in fibrillar region*	0.025*
	StrWidthFD	*Total width of distributed in fibrillar region*	0.024*
	NoXlinkF	*Number of cross-links of all strings in fibrillar region*	0.027*
Count (p<0.05)			*21*
